# Reactive Aggression Affects Response Inhibition to Angry Expressions in Adolescents: An Event-Related Potential Study Using the Emotional Go/No-Go Paradigm

**DOI:** 10.3389/fpsyg.2020.558461

**Published:** 2020-09-30

**Authors:** Lijun Sun, Junyi Li, Gengfeng Niu, Lei Zhang, Hongjuan Chang

**Affiliations:** ^1^ School of Psychology, Xinxiang Medical University, Xinxiang, China; ^2^ School of Psychology, Sichuan Normal University, Chengdu, China; ^3^ School of Psychology, Central China Normal University, Wuhan, China; ^4^ School of Nursing, Xinxiang Medical University, Xinxiang, China

**Keywords:** response inhibition, angry expressions, emotional Go/No-go task, N2, P3, reactively aggressive adolescents

## Abstract

Although it is well established that response inhibition to angry expressions is impaired among reactively aggressive adolescents, the cognitive processes underlying this effect remain unclear. The main goal of our study was to investigate the time course of response inhibition to angry expressions in reactively aggressive adolescents compared to controls. In total, 23 reactively aggressive adolescents and 23 control adolescents were recruited to participate in an event-related potential (ERP) study measuring response inhibition to angry expressions with an emotional Go/No-go paradigm. The results showed that when presented angry or happy expressions, reactively aggressive adolescents showed a smaller No-go P3 effect than the control group. These results indicate that response inhibition to angry expressions in reactively aggressive adolescents is impaired at the later stage of the actual inhibitory control. The characteristics of response inhibition to happy expressions in reactively aggressive adolescents are similar to those in response to angry expressions.

## Introduction

Reactive aggression (also known as impulsive aggression) refers to aggressive behavior in response to perceived threat or provocation and is the main type of aggressive behavior (Berkowitz, 1993). Reactive aggression can have a serious negative impact on the health and social adjustment of individuals, and this may induce a higher risk for internalized problems, such as anxiety and depression, and a higher risk of depressive disorder, substance abuse, and impaired social relationships, thereby increasing the risk of suicide (Hartley et al., 2018). Although reactive aggression exists across different age groups, it is prominent among adolescents. The adolescent years are known to be a sensitive period for the outbreak of aggressive behaviors, especially in the face of provocation or frustration (Jara et al., 2017). Therefore, adolescents’ reactive aggression and the underlying mechanisms of this have been the focus of much psychological and sociological research (McEwen and McEwen, 2017).

Response inhibition involves the ability to inhibit maladaptive behavior (Luijten et al., 2011) and is thought to be one of the core psychological mechanisms contributing to reactive aggression in adolescents. Empirical studies have revealed impaired response inhibition in reactively aggressive adolescents (Zhang et al., 2017; Hecht and Latzman, 2018). Research on response inhibition suggests that response inhibition may be a flexible resource that is usually in a dormant state but can be awakened in particular contexts, such as a hostile situation (Wilkowski and Robinson, 2010). Angry expressions are often used as stimuli to represent hostile situations (Lievaart et al., 2016). Hence, response inhibition to angry expressions in reactively aggressive adolescents has received considerable attention from researchers.

The integrative cognitive model (ICM) of reactive aggression illustrates the internal cognitive mechanism between hostile situations and such aggression (Wilkowski and Robinson, 2010). In particular, the ICM emphasizes the important role of response inhibition. According to the ICM, when facing angry expressions, individuals will interpret these as hostility and demonstrate reduced response inhibition, which eventually leads to reactive aggression. This has also been examined in behavioral and neural studies. Behavioral studies have shown that when presented with angry expressions, response inhibition is impaired in reactively aggressive adolescents (Denson et al., 2011). Brain imaging studies have found that when individuals are presented with angry expressions, orbitomedial prefrontal cortex (OMPFC) reactivity and functional connectivity from limbic system (such as in the caudate nucleus and amygdala) to the OMPFC are significantly and negatively correlated with reactive aggression (Beyer et al., 2015; da Cunha-Bang et al., 2017; Jiang et al., 2018). The OMPFC is associated with response inhibition in hostile situations (Koenigs and Tranel, 2007). Therefore, we speculate that when presented with angry expressions, reactively aggressive adolescents show deficits in response inhibition, resulting from reduced brain reactivity in the OMPFC, and reduced functional connectivity between the OMPFC and limbic system. However, the time course of response inhibition to angry expressions in reactively aggressive adolescents remains unclear.

The study of event-related potentials (ERPs), which have a high temporal resolution, is a useful method to explore the information processing stages. In ERP studies, the emotional Go/No-go paradigm has been frequently used to measure response inhibition to emotional stimuli because of relatively clear and simple cognitive components of this task (Luijten et al., 2014). It is a variant on the classic Go/No-go paradigm. In this task, emotional stimuli are used as the Go or No-go stimuli, and participants are asked to respond to the Go stimuli but not to the No-go stimuli. Studies using ERPs in the emotional Go/No-go task typically demonstrate two major ERP components that are increased during successful inhibition of a dominant response (i.e., are larger on No-Go trials compared to Go trials) and, hence, may represent valuable markers for response inhibition: the N200 and P300 components (Falkenstein et al., 1999). The N2 has a maximum amplitude in the frontal regions at 200–400 ms after the stimulus is presented, and the P3 has a maximum amplitude in the frontocentral regions at 300–700 ms after the stimulus is presented. Compared with the Go trials, the N2 and P3 are remarkably larger in No-go trials. The difference waves (No-go minus Go ERPs) of the N2 (N2d) and P3 (P3d) represent the No-go N2 and No-go P3 effects. The No-go N2 effect is thought to reflect the early stages of conflict monitoring before the correct response is made (Falkenstein et al., 1999). The No-go P3 effect is thought to reflect the later stage of actual inhibitory control of the motor system (Smith et al., 2008). Studies found that compared to adults, adolescents were less able to inhibit unwanted action tendencies in emotional Go/No-go tasks (Hare et al., 2008; Hämmerer et al., 2010). Existing studies reported a decrease of the No-go N2 effect with increasing age in participants aged 10–36 years, and this effect was largest between 10 and 20 years of age (Johnstone et al., 2005). Studies also indicated that facing threatening facial expressions (such as angry or fearful faces), individuals with high trait aggression showed a smaller No-go P3 effect than adolescents with low trait aggression, and response inhibition to facial distress was inversely associated with trait aggression (Fido et al., 2017; Sun et al., 2020). Trait aggression is significantly associated with aggressive behavior (Sherrill et al., 2016), while trait aggression is a personality trait, and aggressive behavior is a kind of behavior of purposeful harm to another person. Aggressive behavior is divided into proactive aggression and reactive aggression. Reactive aggression is characterized by a spontaneous emotion-driven response to perceived provocation, while proactive aggression, on the other hand, is characterized by deliberate, non-emotional behavior to obtain a specified goal (Crick and Dodge, 1996). Response inhibition was found to be related to reactive aggression but not to proactive aggression (Zhang et al., 2017).

Based on these findings, we adopted ERP technology to explore the cognitive processing stages of response inhibition to angry expressions in reactively aggressive adolescents using the emotional Go/No-go paradigm. This is of great significance for revealing the internal mechanisms of reactive aggression and providing both a theoretical foundation and objective biological markers for establishing an early warning of risk system and clinical interventions. We predicted that when participants are presented angry expressions, the No-go N2 and No-go P3 effects in reactively aggressive adolescents would be smaller than in the control group. At the behavioral level, we expected that when presented angry expressions, reactively aggressive adolescents would make more mistakes in the No-go task.

## Materials and Methods

### Participants

According to the effect size of group differences between high and low aggressive individuals in response inhibition in the study of Lievaart et al. (2016; *η*_p_^2^ = 0.38), we adopted the G*Power 3.1 software and set power to 95% and the alpha level to 0.05. The sample size was calculated in each group to be 18. The present study was approved by the Institutional Review Board of Xinxiang Medical University. We recruited 1,000 freshmen (500 females) from a Chinese public university and obtained written informed consent. The Chinese version of the Buss-Perry Aggression Questionnaire (BPAQ) and the Reactive-Proactive Aggression Questionnaire (RPQ) was used to screen reactively aggressive adolescents. In accordance with existing research (Chang et al., 2018), the inclusion criteria were as follows: scores for the BPAQ and reactive aggression being greater than mean + 1σ and the difference between reactive aggression and proactive aggression in the RPQ being greater than mean + 1σ. The inclusion criteria for the control group were scoring <mean + 0.5SD on both the BPAQ and the RPQ. Considering the gender balance, 23 reactively aggressive adolescents (12 female) and 23 control adolescents (12 female) participated. The age range of the sample was 17–20 years (*M*age = 18.33). Data from four participants were discarded from the ERP data analysis, as more than 50% of their epochs included artifacts. All participants had normal or corrected-to-normal vision and were right-handed. After completing the experiment, each participant received an appropriate amount of compensation. No significant difference was found for age between the reactively aggressive group (*M* = 18.43, *SD* = 0.79) and the control group [*M* = 18.22, *SD* = 0.67; *t*(44) = 1.01, *p* = 0.32]. The scores for reactive aggression in the reactively aggressive group (*M* = 12.87, *SD* = 0.97) were significantly higher than in the control group [*M* = 5.13, *SD* = 1.39; *t*(44) = 21.90, *p* < 0.001, *d* = 6.45].

### Questionnaires

The revised Chinese version of the Aggression Questionnaire (AQ; Buss and Perry, 1992; Luo, 2008) was adopted. It is a widely used measure of aggression consisting of 29 items that can be divided into four dimensions: verbal aggression, physical aggression, hostility, and anger. Sample statements from the four dimensions include: “When people disagree with me, I cannot help arguing with them” (verbal); “If someone hits me, I will hit back” (physical); “Sometimes I feel people laughing at me behind my back” (hostility); and “When I get frustrated, I let my anger show” (anger). Participants were asked to respond on a five-point Likert scale ranging from 1 (extremely uncharacteristic of me) to 5 (extremely characteristic of me). A higher score indicates higher aggression. In this study, the AQ showed good internal reliability (*α* = 0.85).

The revised Chinese version of the RPQ (Raine et al., 2006; Zhang et al., 2014) was adopted to measure proactive and reactive aggression. It includes 23 items, which can be divided into two dimensions: proactive aggression (12 items) and reactive aggression (11 items). Sample statements from the two dimensions include: “To get what you want, team up with some people against others” (proactive) and “If someone annoys me, I yell at him” (reactive). The RPQ assesses the frequency with which participants have participated in the behavior described in each item as follows: often (2), sometimes (1), or never (0). In the current study, the proactive aggression and reactive aggression subscales exhibited good internal reliability (*α* = 0.87 and *α* = 0.89).

### Emotional Face Stimuli

Angry and happy faces (*n* = 72 of each) were selected from the Chinese Facial Affective Picture System (CAPS; Wang and Luo, 2005). In total, faces from 36 females and 36 males were used. Before the experiment, 25 adolescents (12 males, 17–20 years) were asked to rate each picture from 1 to 9 on arousal (1 = calming, 9 = arousing) and valence (1 = negative, 9 = positive). A *t*-test showed that the stimuli differed significantly in valence (*t* = 7.99, *p* < 0.001; *M* ± *SD*, angry: 2.91 ± 0.86, happy: 5.17 ± 1.06) but not arousal (*p* > 0.05). All images were consistent in size and contrast.

### Emotional Go/No-Go Task

We used the emotional Go/No-go paradigm to measure response inhibition. Angry and happy expressions were used as frequent Go or infrequent No-go stimuli. Participants were required to respond as soon and accurately as possible to the target stimuli (Go cues) by pressing a button with their index fingers and not to respond to the non-target stimuli (No-go cues). The task was divided into two blocks: anger Go/happiness No-go, happiness Go/anger No-go) with the order of the blocks counterbalanced across participants. Each block contained 72 (30%) No-go trials and 168 (70%) Go trials, which were presented in a pseudorandom order. No-go trials were always preceded by a Go trial to induce pre-potent conflict and motor responses during response inhibition.

**Figure 1:**
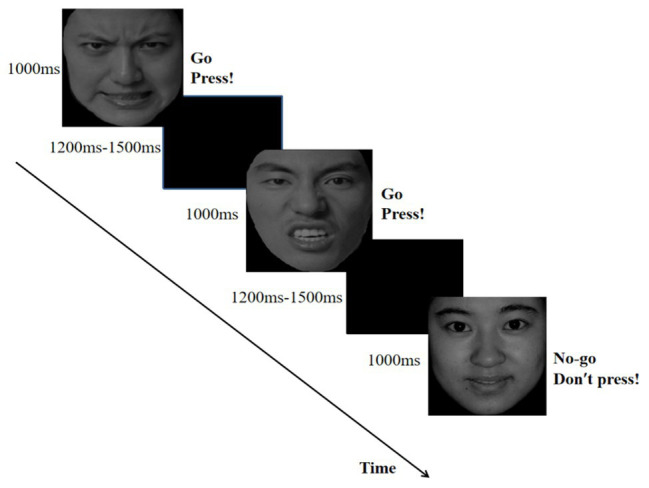
The flow map of emotional Go/No-go task in which angry expressions served as Go cues and happy expressions were No-go cues. Angry and happy faces were selected from the Chinese Facial Affective Picture Systom (Wang and Luo, 2005).

In each trial, a fixation point was presented for 400–600 ms, followed by an emotional face for 1,000 ms and then a blank screen for 1,200–1,500 ms as shown in Figure 1. Each participant completed 480 trials. Between the two blocks, participants could take a short break. Participants performed two short practice blocks before the formal experiment.

### Event-Related Potential Recording and Analysis

Electroencephalographic (EEG) data were recorded from a 64-channel Neuroscan system (Neuroscan SynAmps2; NeuroScan Inc., Sterling, VA, United States) according to the extended international 10/20 system. The reference electrode was placed at the top of the head. Vertical electrooculogram data were recorded with one pair of electrodes placed above and below the left eye. Horizontal electrooculogram data were recorded with another pair of electrodes placed at the outer canthi of both eyes. Electrode impedance was kept below 5 kΩ. The EEGs were recorded using a band-pass filter of 0.05–100 Hz. All signals were sampled at 500 Hz and 32-bit A/D conversion.

EEG data were re-referenced offline to the average of left and right mastoids before further analysis. To remove high-frequency noise, a filter with a band pass of 0.15–30 Hz was used. Independent component analysis was adopted to reject the blinks and eye movement artifacts. In the ERP analysis, trials with a voltage exceeding ±75 μV were eliminated. We computed the stimulus-locked epochs. Each epoch was segmented from 200 ms prior to stimulus onset to 1,000 ms after stimulus onset, and those from 200 ms before stimulus onset served as a baseline.

For the N2 and P3 components, we calculated the base-peak amplitudes of the ERPs. Referring to the existing literature (Lievaart et al., 2018; Sun et al., 2020) and the ERP grand average waveforms, we selected nine electrode sites (F3, Fz, F4, FC3, FCz, FC4, C3, Cz, and C4) for statistical analysis of the N2 (210–420 ms) and P3 (320–720 ms) amplitudes.

### Statistical Analyses

#### Behavioral Analysis

To assess the effects of group, valence, and trial type, we conducted a 2 (group: reactively aggressive group vs. control group) × 2 (emotion: angry expressions vs. happy expressions) × 2 (trial type: Go vs. No-go) repeated-measures ANOVA on error rates. With respect to reaction times (RTs) to correct Go trials, we performed a 2 (group: reactively aggressive group, control group) × 2 (emotion: angry expressions, happy expressions) repeated-measures ANOVA. Response times outside 150–1,500 ms were excluded.

#### ERP Analyses

Repeated-measures ANOVAs (2 × 2 × 2 × 9) were carried out for the N2 and P3 amplitudes with group (reactively aggressive group, control group) as a between-subject factor and trial type (Go, No-go), emotion (angry expressions, happy expressions), and Electrode (nine frontocentral electrodes: F3, Fz, F4, FC3, FCz, FC4, C3, Cz, and C4) as within-subject factors. The Greenhouse–Geisser method was used where appropriate. The Bonferroni method was used for multiple comparisons. Simple effect tests were performed only for interactions including the between-subject factor of group.

#### Correlation Analyses

Referring to the analysis methods of Euser and Franken (2012) and Zhang et al. (2016), Spearman rank correlation was used to analyze the correlation between reactively aggressive scores and ERP indicators in the reactively aggressive group and the control group. The average amplitude of Go/No-go difference waves at the measured electrode sites and the two emotions of anger and happiness were used as EEG indicators for the N2 and P3.

## Results

### Behavioral Performance

The commission and omission error rates for the reactively aggressive group and control group are given in Table 1. Error rate data showed that the main effect of emotion was significant [*F*(1,44) = 10.60, *p* = 0.002, *η* ^2^ = 0.19]. Overall, participants were less accurate in the angry condition (*M* = 16.24%) than the happy condition (*M* = 11.69%).

**Table 1:** Behavioral results for the emotional Go/No-go task for the reactively aggressive group and control group.

Groups	Variables	Emotion			
		Angry		Happy	
		*M*	*SD*	*M*	*SD*
Reactively aggressive group	CE (%)	15.88	1.82	13.16	1.91
	OE (%)	26.07	3.67	13.83	1.74
	Go RT (ms)	500.21	14.98	509.06	11.17
Control group	CE (%)	10.21	1.82	11.41	1.91
	OE (%)	12.81	3.67	8.33	1.74
	Go RT (ms)	539.76	14.98	522.74	11.17

CE, percentage of commission errors; OE, percentage of omission errors; RT, reaction time for correct Go trials; ms, milliseconds.

For the effect of reactive aggression, there was a main effect of group [*F*(1,44) = 11.04, *p* = 0.002, *η* ^2^ = 0.20], with more error rates in the reactively aggressive group than the control group (*M* error rates were 17.24 vs. 10.69%, respectively). The interaction between group and emotion [*F*(1,44) = 4.36, *p* = 0.043, *η* ^2^ = 0.09] was significant. In the angry condition, the reactively aggressive group was less accurate (*M* = 20.98%) than the control group (*M* = 11.51%; *p* = 0.001). In the happy condition, the reactively aggressive group was also less accurate than the control group (*M* = 13.50 and *M* = 9.87%, respectively; *p* = 0.079). The interactions between group and trial type and between group and emotion and trial type were not significant (*p*s > 0.05).

The RTs for Go trials in the reactively aggressive group and control group are given in Table 1. Reaction time data indicated that the main effects of group and emotion and the interaction between group and emotion were not significant (*p*s > 0.05).

### Raw ERP Waveforms

**Figure 2:**
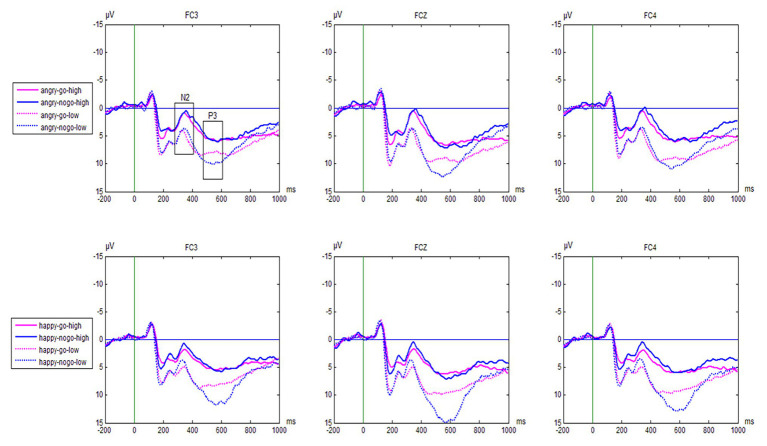
Grand average waveforms from the three electrode sites (FC3, FCz, FC4, respectively) evoked by the valence and trial type in the emotional Go/No-go task as a function of group (reactively aggressive group vs. control group).

Grand average waveforms are shown in Figure 2. The N2 and P3 amplitudes for the emotional Go/No-go task for the reactively aggressive group and control group are shown in Table 2.

**Table 2:** N2 and P3 amplitudes for the emotional Go/No-go task for the reactively aggressive group and control group.

Valence	Trial type	Reactively aggressive group (*n* = 23)				Control group(*n* = 23)			
		N2		P3		N2		P3	
		*M*	*SD*	*M*	*SD*	*M*	*SD*	*M*	*SD*
Angry	Go	−0.50	0.68	7.80	0.72	2.84	0.68	11.02	0.72
	No-go	−0.89	0.76	8.05	0.82	2.36	0.76	14.43	0.82
Happy	Go	0.33	0.74	6.81	0.82	3.15	0.74	11.10	0.82
	No-go	−1.31	0.75	8.02	0.84	2.18	0.75	12.36	0.84

N2 and P3 are mean amplitudes in microvolts averaged across six recorded frontocentral scalp sites (F3, Fz, F4, FC3, FCz, FC4, C3, Cz, and C4).

#### N2

We observed a significant main effect of trial type [*F*(1,44) = 18.87, *p* < 0.001, *η* ^2^ = 0.30], with larger amplitudes to No-go trials than Go trials (*M* = 0.59 ± 0.51 vs. 1.45 ± 0.49 μV, respectively). We also found a significant main effect of Electrode [(*F*(8,352) = 10.93, *p* < 0.001, *η* ^2^ = 0.20], indicating that N2 amplitudes, which were largest at F4 electrode sites (0.07 ± 0.48 μV), were mainly distributed in prefrontal areas.

For the effects of reactive aggression, a significant main effect of group was found [*F*(1,44) = 10.97, *p* = 0.002, *η* ^2^ = 0.20]. Overall, the reactively aggressive group showed significantly larger N2 amplitudes than the control group (−0.59 ± 0.69 vs. 2.63 ± 0.69 μV, respectively). Notably, the interactions between group, emotion, trial type, and electrodes reached significance [*F*(8,352) = 1.99, *p* = 0.047, *η* ^2^ = 0.43]. To analyze this effect further, we analyzed the Go/No-go difference waves to determine the difference in response inhibition between the reactively aggressive group and the control group. No other main effect or group-related interaction effect was observed.

#### P3

We observed a significant main effect of trial type [*F*(1,44) = 11.92, *p* = 0.001, *η* ^2^ = 0.18], with larger P3 amplitudes to No-go trials than Go trials (10.72 ± 0.55 vs. 9.18 ± 0.52 μV, respectively). We also found a significant main effect of emotion [*F*(1,44) = 11.92, *p* = 0.001, *η* ^2^ = 0.18], with larger amplitudes to angry expressions (10.33 ± 0.53 μV) relative to happy expressions (9.58 ± 0.56 μV). Furthermore, a significant main effect of Electrode emerged [*F*(8,352) = 18.74, *p* < 0.001, *η* ^2^ = 0.26], with larger amplitudes at Cz and FCz (12.40 ± 0.74 and 10.93 ± 0.61 μV) than at the other electrodes (all *p*s < 0.01).

For the effects of reactive aggression, we found a significant main effect of group [*F*(1,44) = 18.68, *p* < 0.001, *η* ^2^ = 0.30], with smaller P3 amplitudes in the reactively aggressive group than the control group (7.68 ± 0.74 vs. 12.22 ± 0.74 μV, respectively). The interaction between group and trial type [*F*(1,44) = 9.90, *p* = 0.003, *η* ^2^ = 0.18] was significant. A simple effects test showed that the No-go amplitude was remarkably larger than the Go amplitude (10.10 ± 0.55 vs. 9.12 ± 0.59 μV, respectively) in the control group, and there was also a significant difference between the two amplitudes in the reactively aggressive group (11.09 ± 0.62 vs. 9.18 ± 0.57 μV). The interaction between group, trial type, and electrode was significant [*F*(8,352) = 2.77, *p* = 0.035, *η* ^2^ = 0.06]. Simple effects tests found that at sites F3, Fz, FC4, and CZ, the No-go amplitude in the control group was larger than the Go amplitude (*p*s < 0.01), while there was no significant difference between the amplitudes in the reactively aggressive group (*p*s > 0.05).

Most interestingly, the interaction between group, emotion, and trial type [*F*(1,44) = 18.04, *p* < 0.001, *η* ^2^ = 0.29] was significant. Simple effects tests showed that in the angry condition, the No-go amplitude (14.43 ± 0.82 μV) in the control group was significantly larger than the Go amplitude (11.02 ± 0.72 μV; *p* = 0.003), whereas no significant differences were found in the reactively aggressive group (8.05 ± 0.82 vs. 7.80 ± 0.72 μV, respectively; *p* = 0.175). In the happy condition, the No-go amplitude (12.36 ± 0.84 μV) in the control group was significantly larger than the Go amplitude (11.10 ± 0.82 μV; *p* < 0.001). We also found a significant difference between the No-go and Go amplitudes in the reactively aggressive group (8.02 ± 0.84 vs. 6.81 ± 0.82 μV, respectively, *p* < 0.001).

### Go/No-Go Difference ERP Waveforms

As mentioned above, to analyze the interaction between group, emotion, trial type, and Electrode on the N2 component and the interaction between group, emotion, and trial type on the P3 component more clearly, the Go amplitude was subtracted directly from the No-go amplitudes on each emotion condition at each electrode.

#### N2 Difference Wave

There was a significant emotion main effect for the N2 [*F*(1, 44) = 5.11, *p* = 0.029, *η* ^2^ = 0.10]. The N2 amplitudes were larger in the happy condition (−1.30 ± 0.32 μV) than the angry condition (−0.43 ± 0.22 μV).

Regarding the effects of reactive aggression, we also detected a marginally significant group, emotion, and electrode interaction effect [*F*(8,352) = 1.99, *p* = 0.095, *η* ^2^ = 0.04]. Simple effects tests indicated that at all electrode sites in the angry condition, no remarkable group differences were apparent (*p*s > 0.05). In the happy condition, no remarkable differences between the reactively aggressive group and the control group were found (*p*s > 0.05). Meanwhile, we found that at the F3, Fz, F4, FC3, and FCz electrode sites, happy expressions elicited larger amplitudes as compared to angry expressions in the reactively aggressive group (*p*s < 0.05), while there was no significant emotion difference in the control group (*p*s > 0.05).

#### P3 Difference Wave

The analysis of the P3 difference wave indicated a significant Electrode main effect. FCz (2.46 ± 0.21 μV) and F_Z_ (2.19 ± 0.31 μV) elicited larger amplitudes than the other electrode sites (*p*s < 0.05).

For the effects of reactive aggression, we found a group main effect [*F*(1,44) = 9.90, *p* = 0.003, *η* ^2^ = 0.18]. Overall, the reactively aggressive group demonstrated significantly smaller P3 amplitudes as compared to the control group (0.76 ± 0.35 vs. 2.31 ± 0.35 μV, respectively). We also found a significant interaction between group and electrode [*F*(8,352) = 2.77, *p* = 0.035, *η* ^2^ = 0.06]. Simple effects tests indicated that at each electrode site, the reactively aggressive group showed smaller amplitudes relative to the control group.

**Figure 3:**
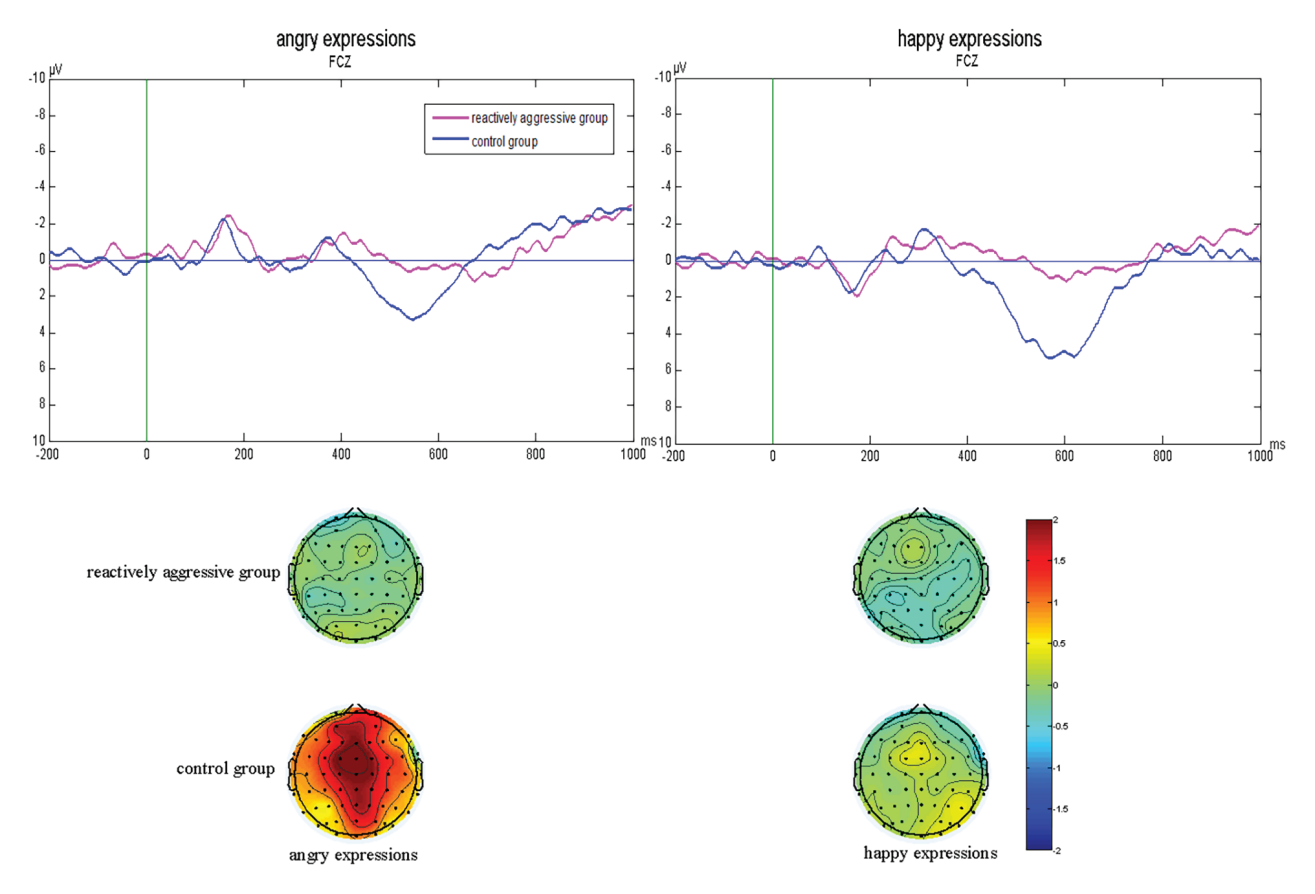
P3 difference waves of No-go subtracted from Go trials at the FCz site and corresponding scalp topography of the two groups in angry and happy condition.

Most interestingly, there was also a significant group and emotion interaction effect [*F*(1,44) = 18.04, *p* < 0.001, *η* ^2^ = 0.29]. Simple effects tests showed that in the angry condition, the reactively aggressive group demonstrated significantly smaller P3 amplitudes as compared to the control group (0.24 ± 0.40 vs. 3.40 ± 0.40 μV, respectively). In the happy condition, the reactively aggressive group also demonstrated significantly smaller P3 amplitudes as compared to the control group (1.21 ± 0.48 vs. 1.26 ± 0.48 μV, respectively). P3 difference waves of No-go subtracted from Go trials at the FCz site and corresponding scalp topography of the two groups in angry and happy conditions are shown in Figure 3. Meanwhile, we found that angry expressions (3.40 ± 0.40 μV) elicited larger amplitudes as compared to happy expressions (1.26 ± 0.48 μV) in the control group (*p* = 0.079), while there was no significant emotion difference in the reactively aggressive group (3.40 ± 0.40 vs. 1.21 ± 0.48 μV; *p* = 0.364).

### Correlations Between Reactively Aggressive Scores and Go/No-Go Difference Waves

The correlation analysis found that reactively aggressive scores were negatively related to the amplitudes of the P3 Go/No-go difference waves (*r* = −0.46, *p* = 0.002). There was no relation between reactively aggressive scores and the amplitudes of the N2 Go/No-go difference wave (*r* = −0.02, *p* = 0.899).

## Discussion

In the present study, we used high-resolution ERP technology to explore the cognitive processing stages of response inhibition to angry expressions in reactively aggressive adolescents using an emotional Go/No-go paradigm. Results showed that, overall, participants showed larger N2 and P3 No-go amplitudes than Go amplitudes, confirming that our task was effective in creating a dominant response that is hard to suppress (Euser and Franken, 2012). With respect to the behavioral findings, we found that when presented with angry expressions, reactively aggressive adolescents made the same mistakes as the control group in the No-go task, which was not in line with our expectations. With respect to the neurophysiological findings, when presented angry expressions, reactively aggressive adolescents showed a smaller No-go P3 effect than the control group, which was consistent with some of our expectations. In addition, when presented with happy expressions, these adolescents showed a smaller No-go P3 effect, which indicated that happy faces were as likely to be viewed as hostile ones.

The behavioral analyses indicated that in the anger No-go condition, there was no significant difference between the reactively aggressive adolescents and the control group in error rates. This may be because the Go/No-go task was relatively easy for participants. It should be noted that when presented angry expressions, reactively aggressive adolescents showed shorter reaction times and more commission errors compared to the control group. This trend is in line with the idea that reactively aggressive adolescents show impaired response inhibition to angry expressions and is consistent with findings from previous studies indicating response inhibition deficits in reactively aggressive individuals in non-emotional tasks (Chen et al., 2008; Feilhauer et al., 2012).

Previous studies using non-emotional Go/No-go tasks have found that the N2 Go/No-go difference wave is negatively correlated with impulsivity in aggression in juvenile violent offenders, and impulsive violent offenders demonstrate a smaller amplitude in the N2 Go/No-go difference wave relative to matched controls (Chen et al., 2005; Zhang et al., 2017). The present study recruited participants from the normal population and used the emotional Go/No-go paradigm. We found that during the N2 stage, when presented angry expressions, reactively aggressive adolescents showed the same No-go N2 effect as the control group. The correlation analyses also found that there were no correlations between reactively aggressive scores and the amplitudes of the N2 Go/No-go difference wave, further supporting the ERP results. This is consistent with our previous studies finding that in response to threatening facial expressions, N2 No-go effects are not correlated with trait aggression (Sun et al., 2020). The present results are a useful supplement to the existing research. Moreover, the results in the N2 difference wave showed that angry expressions elicited the same amplitude as happy expressions in the control group, which is also consistent with existing research (Yu et al., 2009). Meanwhile, angry expressions elicited a smaller amplitude as compared to happy expressions in the reactively aggressive group. The N2 difference wave is considered to be an indicator of response conflict and attentional engagement. Angry expressions are often processed preferentially or automatically compared to happy expressions in reactively aggressive adolescents (Qiu et al., 2016; Gagnon and Rochat, 2017). The psychological processing of the response conflicts and attentional engagement were likely to be reduced for angry expressions as compared to happy expression because the brain automatically diverted attention to the angry expressions.

Previous studies using Go/No-go tasks have found that there are no significant differences in the P3 No-go effect between impulsive violent offenders and control groups, and impulsiveness is not related to reduced activation in brain areas associated with response inhibition, such as the right orbital frontal cortex and ventromedial prefrontal cortex in high-risk people (Chen et al., 2005; Brown et al., 2015). This suggests that response inhibition in high-risk groups with high impulsiveness is not impaired. Different from these special groups, healthy individuals with high impulsiveness show a remarkably reduced P3 No-go effect compared to individuals with less impulsiveness (Ruchsow et al., 2008). This suggests that response inhibition in healthy individuals with high impulsiveness is impaired. Reactively aggressive adolescents are highly impulsive healthy individuals. The present study using the emotional Go/No-go paradigm showed that during the P3 stage, reactively aggressive teenagers showed a smaller No-go P3 effect than the control group. This suggests that response inhibition in reactively aggressive adolescents is impaired, which is consistent with previous studies. Furthermore, our previous studies found that facing threatening facial expressions, individuals with high trait aggression showed a smaller No-go P3 effect than individuals with low trait aggression (Sun et al., 2020). Trait aggression is significantly associated with aggressive behavior (Sherrill et al., 2016), while trait aggression is a personality trait, and aggressive behavior is a kind of behavior of purposeful harm to another person. On this basis, this study took reactively aggressive adolescents as the research object to explore the cognitive processing stages of response inhibition to angry expressions. We found that when presented angry expressions, reactively aggressive adolescents showed a smaller No-go P3 effect than the control group. Topographic map results also showed that when participants faced angry expressions, activation in the P3 differential wave topographic map in the reactively aggressive group was lower than that in the control group. These indicate that when facing angry expressions, the later stage of actual inhibitory control of the motor control system is disrupted in reactively aggressive adolescents. The present results are a useful supplement to our previous studies and are consistent with existing studies using fMRI technology (Beyer et al., 2015; da Cunha-Bang et al., 2017; Jiang et al., 2018). According to the ICM, when presented angry expressions, reactively aggressive adolescents engage in more hostile interpretations, thereby impairing the later stage of response inhibition (Wilkowski and Robinson, 2010). The correlation analysis found that reactively aggressive scores were negatively correlated with amplitudes of the P3 Go/No-go difference wave, which further supports the ERP results.

Behavioral studies have shown that trait aggression is related to impaired response inhibition to angry expressions but not happy ones (Denny and Siemer, 2012). However, we found that when presented happy expressions, the reactively aggressive adolescents showed smaller No-go P3 effects than the control group. This suggests that response inhibition to happy expressions in reactively aggressive adolescents is also impaired in the later stage of the actual inhibitory control of the motor system in the premotor cortex. The dual competition model (Pessoa, 2009) assumes that cognitive resources are limited. In particular, reactively aggressive adolescents have a hostile interpretation bias, and they tend to view many nonthreatening situations (such as happy expressions) as threatening (Lake et al., 2014; Bockstaele et al., 2020), which impedes the later stage of response inhibition through reducing the availability of attentional resources. The results of the P3 difference wave also showed that the response inhibition to angry expressions in reactive aggressive adolescents was the same as happy expressions, which provides strong support for the above explanation. Together, these findings indicate that when presented angry expressions, response inhibition in reactive aggressive adolescents is impaired at the later stage of actual inhibitory control of the motor system. Characteristics of response inhibition to happy expressions in reactively aggressive adolescents are similar to those of angry expressions.

There were several limitations to our study. First, only positive emotions were used as the control to explore the response inhibition to angry expressions in reactively aggressive adolescents. In the future, negative expressions (such as fear) should be used as the control to further explore whether the response inhibition to angry expressions in reactively aggressive adolescents has emotion specificity. Second, individuals usually display both proactive and reactive aggression to varying degrees. Thus, individual aggression can be divided into proactive aggression, reactive aggression, proactive–reactive aggression, and non-aggression. This study only investigated the differences in response inhibition to angry expressions between a reactively aggressive group and a control group, and future studies should investigate and compare the differences in response inhibition to angry expressions between the four possible groups. Additionally, we only selected freshmen as research participants. In the future, high school teenagers should be included to make the findings more generalizable. Despite these limitations, our study is important in revealing an internal mechanism of reactive aggression and providing a theoretical foundation as well as objective biological markers for establishing an early warning system of risk and clinical interventions.

In conclusion, our results indicate that response inhibition to angry expressions in reactively aggressive adolescents is impaired in the later stage of the actual inhibitory control of the motor system in the premotor cortex. The characteristics of response inhibition to happy expressions in reactively aggressive adolescents are similar to those for angry expressions.

## Data Availability Statement

The raw data supporting the conclusions of this article will be made available by the authors, without undue reservation.

## Ethics Statement

The studies involving human participants were reviewed and approved by the Institutional Review Board of the Xinxiang Medical University. Written informed consent to participate in this study was provided by the participants’ legal guardian/next of kin.

## Author Contributions

LS: conceptualization, data curation, investigation, and methodology. LZ: formal analysis and writing – review and editing. JL: resources. LS and HC: supervision. LS, JL, and GN: writing – review and editing. All authors contributed to the article and approved the submitted version.

### Conflict of Interest

The authors declare that the research was conducted in the absence of any commercial or financial relationships that could be construed as a potential conflict of interest.
